# Exonic Variants Associated with Development of Aspirin Exacerbated Respiratory Diseases

**DOI:** 10.1371/journal.pone.0111887

**Published:** 2014-11-05

**Authors:** Seung-Woo Shin, Byung Lae Park, HunSoo Chang, Jong Sook Park, Da-Jeong Bae, Hyun-Ji Song, Inseon S. Choi, Mi-Kyeong Kim, Hea-Sim Park, Lyoung Hyo Kim, Suhg Namgoong, Ji On Kim, Hyoung Doo Shin, Choon-Sik Park

**Affiliations:** 1 Genome Research Center for Allergy and Respiratory Diseases, Division of Allergy and Respiratory Medicine, Soonchunhyang University Bucheon Hospital, Bucheon, Republic of Korea; 2 Department of Genetic Epidemiology, SNP Genetics Inc., Seoul, Republic of Korea; 3 Department of Interdisciplinary Program in Biomedical Science Major Graduate School of Soonchunhyang University, Asan, Republic of Korea; 4 Department of Allergy, Chonnam National University Medical School and Research Institute of Medical Sciences, Gwangju, Republic of Korea; 5 Division of Allergy, Department of Internal Medicine, Chungbuk National University, Cheongju, Republic of Korea; 6 Department of Allergy & Clinical Immunology, Ajou University Hospital, Suwoon, Republic of Korea; 7 Department of Life Science, Sogang University, Seoul, Republic of Korea; National Taiwan University, Taiwan

## Abstract

Aspirin-exacerbated respiratory disease (AERD) is one phenotype of asthma, often occurring in the form of a severe and sudden attack. Due to the time-consuming nature and difficulty of oral aspirin challenge (OAC) for AERD diagnosis, non-invasive biomarkers have been sought. The aim of this study was to identify AERD-associated exonic SNPs and examine the diagnostic potential of a combination of these candidate SNPs to predict AERD. DNA from 165 AERD patients, 397 subjects with aspirin-tolerant asthma (ATA), and 398 normal controls were subjected to an Exome BeadChip assay containing 240K SNPs. 1,023 models (2^10^-1) were generated from combinations of the top 10 SNPs, selected by the *p*-values in association with AERD. The area under the curve (AUC) of the receiver operating characteristic (ROC) curves was calculated for each model. SNP Function Portal and PolyPhen-2 were used to validate the functional significance of candidate SNPs. An exonic SNP, exm537513 in *HLA-DPB1,* showed the lowest p-value (*p* = 3.40×10^−8^) in its association with AERD risk. From the top 10 SNPs, a combination model of 7 SNPs (exm537513, exm83523, exm1884673, exm538564, exm2264237, exm396794, and exm791954) showed the best AUC of 0.75 (asymptotic *p*-value of 7.94×10^−21^), with 34% sensitivity and 93% specificity to discriminate AERD from ATA. Amino acid changes due to exm83523 in *CHIA* were predicted to be “probably damaging” to the structure and function of the protein, with a high score of ‘1’. A combination model of seven SNPs may provide a useful, non-invasive genetic marker combination for predicting AERD.

## Introduction

Aspirin (acetylsalicylic acid, ASA) - hypersensitivity refers to de­velopment of aspirin-exacerbated respiratory diseases (AERD), and ocular and skin manifestations following ingestion of aspirin or other nonsteroidal anti-inflammatory drugs (NSAIDs) [1]. The prevalence of aspirin hypersensitivity in adult asthmatics varies widely depending on whether it is identified by clinical history alone or after challenge with ASA [2]. Based on patients’ histories alone, the incidence of AERD in adults is 38%, but this percentage increases three-fold after ASA challenge via the oral or bronchial route [3]. It is noteworthy that approximately one-fifth of patients are unaware that they suffer from aspirin intolerance [2]. Thus, identification of aspirin hypersensitivity, especially in hidden cases, is essential to avoid serious bronchospasm attacks in asthmatics.

Diagnosis of AERD can be established with certainty only by provocation tests such as Oral aspirin challenge (OAC). However, OAC is a time-consuming procedure, and in some cases, serious complications can occur. Thus, the development of non-invasive diagnostic methods is necessary to prevent the unexpected complications of aspirin use in susceptible patients.

Fewer than 100 genetic variants have been identified to date. They include biologically plausible genes responsible for the over- or under-production of critical modulators in the metabolism of arachidonic acids, and their receptors include *LTC4S* [4] *ALOX5* [5], *CYSLT1R* [6], *CYSLT2R* [7], *PTGER* [8]–[10], *TBXAS1* [11], and *TBXA2R* [12]. as well as those in the immune response and inflammatory pathways, including DPB1*0301 [13], *IL-4* [14], *T-Box* [15], *FcepsilonR1* [16], [17], *TLR3* [18], *NLRP3* [19], *ADAM33* [20], *ADORA1* [21], *ACE* [22], *CRTH2* [23], *PPARG* [24], *KIF3A* [25], *SLC6A12* [26], *SLC22A2* [27] and *CACNG6* [28]. The statistical significance of these SNPs, however, was marginal, with an odds ratio (OR) ranging from 0.5∼2.0 [29].

Our previous genome-wide association studies (GWAS) using 100,000 SNPs, revealed that rs7275857 in exon 2 of *CEP68* and rs1042151 in *HLA-DPB1* (Met105Val) were associated with AERD with an OR of 2.63 [30] and 2.40 [31] respectively. The limiting factors of these studies included the analysis of common variants with >5% minor allele frequency (MAF) and minimal coverage of exonic SNPs [32], [33]. Thus, recent genetic studies have focused on the possible contributions of rare variants (MAF <1%) that confer a substantial risk of disease. On the basis of growing information on these rare variants, a new Human Exome BeadChip (Illumina Inc., San Diego, CA, USA) has been developed to cover the putative functional exonic variants selected from >12,000 individual exome- and whole-genome sequences [34]. We performed a GWAS using the HumanExome BeadChip v1.1 (Illumina Inc.) to identify new genetic variants and, in particular, exonic variants associated with the risk of AERD, and we evaluated the diagnostic potential of these candidate SNPs to predict AERD in Korean patients with asthma.

## Materials and Methods

### Ethics statement

Written informed consent was obtained from all study participants. For minors/children, it obtained from next of kin, caretakers, or guardians by written form. The protocol was approved by the Soonchunhyang Bucheon Hospital’s ethics committee (SCHBC_IRB_05_02 and schbc-biobank-2012-004). Genomic DNA from all the ethnic Korean study subjects was obtained from a biobank at Soonchunhyang Bucheon Hospital.

### Subjects

Study subjects were recruited from the Asthma Genome Research Center consisting of four university hospitals. They were ethnic Koreans. Asthma was diagnosed by physicians. All patients had a history of dyspnea and wheezing in the previous 12 months, plus one of the following: (1) >15% increase in forced expiratory volume in 1 s (FEV1) or >12% increase plus 200 mL following inhalation of a short-acting bronchodilator, (2) <10 mg/mL PC20 methacholine and (3) >20% increase in FEV1 following 2 weeks of treatment with inhaled or systemic corticosteroids and they met the criteria for asthma according to the Global Initiative for Asthma (GINA) guidelines [35]. Total IgE was measured by the CAP system (Pharmacia Diagnostics, Uppsala, Sweden). Twenty-four commonly inhaled allergens were used for a skin-prick test. Atopy was defined as a wheal reaction with a diameter of 3 mm or greater than the reaction to histamine. Questions related to five specific elements of aspirin hypersensitivity (dyspnea, wheezing, nasal blockage, skin eruption, and loss of consciousness after aspirin ingestion) were incorporated in the questionnaires. Oral aspirin challenge (OAC) was performed to all of the asthmatics. OAC was performed with increasing doses of aspirin using the methods previously described [36], [37]. Patients having a history of aspirin hypersensitivity were given a dose of 30 mg and those with no history started 100 mg of aspirin orally. Symptoms, external signs (urticaria, angioedema, and rhinorrhea), blood pressure, and FEV1 were documented every 30 min for a period of 2 h. In the absence of any symptoms or signs suggestive of an adverse reaction, 100 mg of aspirin were administered and the same measurements were repeated every hour, with doses of 450 mg given until the patient developed a reaction. The test was deemed negative if no reaction occurred within 4 h after the final dose. Aspirin-induced bronchospasm was calculated as the pre-challenge FEV1 minus the post-challenge FEV1 divided by the pre challenge FEV1. OAC reactions were categorized into two groups as follows: (1) 15% or greater decreases of FEV1 or appearance of naso-ocular reactions (AERD); and (2) decreases of less than 15% of FEV1 without naso-ocular and cutaneous reactions [aspirin-tolerant asthma (ATA)]. Normal controls were spouses of the subjects or general populations, gave negative answers to a screening questionnaire for respiratory symptoms and showed >70% of FEV_1_/FVC, and a normal chest X-ray.

### Genome – wide exon SNP genotyping and quality control

Approximately 200 ng of genomic DNA was used to genotype each sample on the Illumina HumanExome v1.1 BeadChip (Illumina, Inc., San Diego, CA, USA). Samples were processed according to the Illumina Infinium assay manual. Briefly, each sample was whole-genome amplified, fragmented, precipitated, and resuspended in an appropriate hybridization buffer. Denatured samples were hybridized on a prepared HumanExome v1.1 BeadChip for a minimum of 16 hours at 48°C. Following hybridization, the beadchips were processed for the single-base extension reaction, stained, and imaged on an Illumina Bead Array Reader. Normalized bead intensity data obtained for each sample were loaded into the GenomeStudio software (Illumina, Inc.), which converted fluorescent intensities into SNP genotypes. SNP clusters for genotype calling were examined for all SNPs using the GenomeStudio software. For quality control, only SNPs that were genotyped in more than 98% of samples were included in the further analysis, and SNPs that met over 0.97 of call rate and polymorphic in current subjects were retained. Cluster plots were then assessed visually, and SNPs with poor cluster quality were removed. To examine population substructure, the population stratification analyze was performed.

### Selection of candidate SNPs to predict AERD

First, the case-control study was applied to the exome chip to select the candidate 10 SNPs with AERD. The best combination of the 10 SNPs for discriminating between the AERD and ATA, multiple logistic regressions were performed using ‘R’ software (ver. 2.13.1; http://www.r-project.org/). ROC curves were obtained for all models, and the area under the curve (AUC) was calculated for each model in order to select the best combination of marker SNPs for discriminating AERD from ATA. The 5-fold cross-validation was applied to validate the best prediction model obtained by using multiple logistic regressions.

### The functional implication and structure effect of the candidate SNPs

To find the functional significance of SNPs, SNP functional Portal was used [38]. It was designed to be a clearing house for all public domain SNP functional annotation data, as well as in-house functional annotations derived from different data sources. It contains SNP functional annotations for genomic elements, transcription regulation, protein function, pathway, disease and population genetics.

Because the most SNPs in exome chip were located in the exon region, the structure stability test for the selected SNPs was performed using PolyPhen-2 [39]. The Polyphen uses protein sequences from UniProtKB/UniRef100 Release 2011_12 (14-Dec-2011), structures from PDB/DSSP Snapshot 03-Jan-2012 (78,304 entries) and UCSC MultiZ multiple alignments of 45 vertebrate genomes with hg19/GRCh37 human genome (08-Oct-2009). The result for the structure stability was presented as a probability from ‘0’ to ‘1’. ‘0’ means that there is no impact of amino acid substitutions on the structure and function of human proteins and ‘1’ means much impact on the structure and function.

### Statistical analyses

Statistical analyses were performed using R software (ver. 2.13.1; http://www.r-project.org/). For sex, smoking and atopy variable that were summarized as frequencies, the fisher’s exact test was applied. Because age, onset of asthma, decline (%) of FEV, BMI, Blood eosinophil (%), FEV1% predicted, FVC% predicted and PC20 histamine (mg/mL) didn’t satisfy the normality assumption, they were expressed as medians and ranges and Mann-Whitney U test was applied. The statistical significance was defined as *p*-value<0.05. To examine the association between genotypes and AERD and to calculate odds ratios (ORs) per allele, allelic tests, genotype tests, and Cochran-Armitage trend tests were used. A quantile-quantile plot was used to evaluate overall significance of the genome-wide exon association results and the impacts of population stratification.

## Results

### Clinical characteristics of the study populations

Total 560 asthmatics (165 AERD and 397 ATA) and 398 normal controls were included. Age, onset age of asthma, smoking status, decline (%) of FEV1 by aspirin provocation, BMI, FEV1, % predicted and PC20 methacholine were significantly different between the two groups (*p*-value<0.01). (Table 1).

**Table 1:** Clinical profiles of the study subjects.

	AERD	ATA	NC
Number of subjects (*n*)	165	397	398
Age	41 (17–72)* ^,^ **	48 (15–77)**	57 (11–86)
Age at asthma onset	34 (1–68)*	41 (1–76)	N/A
Sex (male/female)	165 (63/102)	397 (120/277)**	295 (173/225)
Smoker/Ex-smoker (%)	12.12/6.06*	8.06/14.60**	18.34/15.32
Decline (%) of FEV_1_ by aspirin provocation	28.10 (17.6–100)*	4 (−15.00–14.7)	ND
FEV_1_, % predicted	82.5 (68–110)* ^,^ **	78 (68–130)**	105.00 (75–168)
PC20 methacholine (mg/mL)	1.00 (0.07–25.00)* ^,^ **	1.85 (0.01–25.00)**	25.00 (13.6–25.00)
Positive skin prick test (%)	50.03**	50.88**	23.86
Body mass index (kg/m^2^)	23.69±4.27* ^,^ **	24.45±3.74	24.34 (14.8–36.21)

All data are presented as medians (range) or means ± standard deviation.

*: *p*-value<0.01 vs. ATA.

**: *p*-value<0.01 vs. NC.

### Genome – wide exon SNP genotyping and quality control

**Figure 1:**
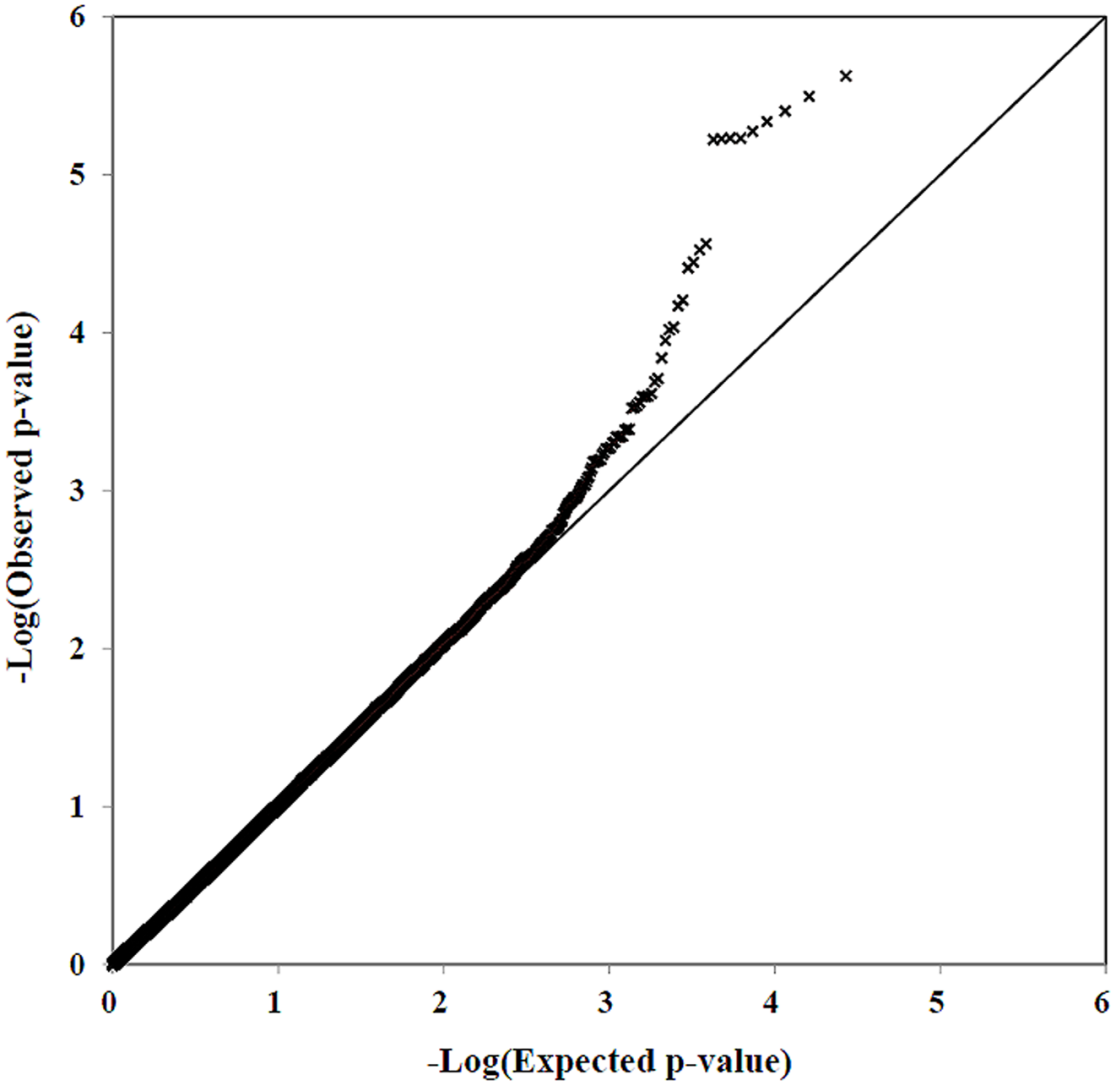
QQ-plot analysis of 54,555 SNPs between for 165 AERD vs. 397 ATA.

Firstly, we defined the cut-off value of call rate as 97%. Then genotype clusters for 500 variants showing call rate nearby 97% (500 above 97%) were plotted using GenomeStudio (Illumina, Inc., San Diego, CA, USA) software optimized for Korean population. In this study, among 242,985 SNPs displayed on the chip, 1,132 SNPs with call rate <97% and with poor cluster quality were removed. Among the remaining 241,853 SNPs, we checked MAF and removed additional 187,298 SNPs which were monomorphic in our study subjects. Finally, a total 54,555 autosomal SNPs were used for further statistical analysis. The overall call rate for all SNPs was 99.91% and only 6,878 SNPs among the 545,555 SNPs (12.6%) were found to have a MAF<1%. The result of the MDS (multidimensional scaling) plot analysis showed there was no the population substructure (Figure S1). These SNPs were then used for association analysis with AERD under the additive mode using logistic regression and because age, smoking and BMI were significantly different between the two groups in the present and other studies [36], [40], [41], and because female are predominant [42], [43] and Atopy is lower in AERD than general asthma [44]. All these parameters were used as covariate. The comparison between the observed and the expected association *p*-values in the quantile-quantile (Q-Q) plot revealed that λ value was 1.0013 (Fig. 1). The data discussed has been deposited in NCBI’s Gene Expression Omnibus [45] and is accessible through GEO Series accession number GSE61129 (http://www.ncbi.nlm.nih.gov/geo/query/acc.cgi?acc=GSE61129).

### Association of the exonic SNPs with the risk of AERD

**Figure 2:**
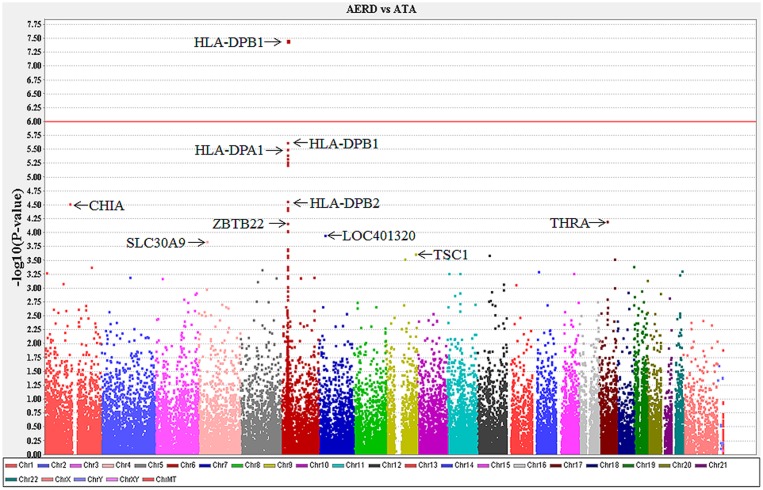
Manhattan plot of the *p*-values of 54,555 autosomal SNPs in association with AERD.

The *p*-values of all SNPs in association with the risk of AERD under the additive mode are presented in a Manhattan plot (Fig. 2). The exm537513 on *HLA-DPB1* showed the lowest *p*-value of 3.4×10^−8^ (OR: 3.28,) in association with AERD. The *p*-value remained significant after multiple comparisons using Bonferroni’s correction (*p* = 0.0082).

The top 100 SNPs are presented according to their p-values in Table S1. Sixteen of the top 20 SNPs were located on chromosome 6. Among them, six SNPs were exonic and located within the *HLA* genes (three in *HLA-DPB1*, two in *HLA-DPA1* and one in *HLA-DPB2*). The remaining 10 SNPs were located at/near a pseudogene or an intron or within an intergenic area (Table S1). There was strong linkage disequilibrium (LD) between the six exonic SNPs in the *HLA* genes, although not complete LD (Table S2). AUCs for the six SNP combinations (2^6^− 1) were calculated by multiple logistic regression (Table S3). A combination of four SNPs (exm537513, exm537522, exm-rs3097671, and exm-rs3129294) had the best AUC value of 0.629.

To determine the top 10 SNPs, the top six SNPs sorted by *p*-value were added to the four *HLA* SNPs (Table 2). The *p*-values of the top 10 SNPs ranged from 3.4×10^−8^ 2.4×10^−4^ with ORs ranging from 0.13∼13.61 (Table 2). Among them, *THRA* and *SLC30A9* were rare variants with a frequency <1% in one or more of the study groups. ORs of these two SNPs were significantly higher or lower (*THRA*, 13.6 and *SLC30A9*, 0.13) compared with those of the other common variants with a MAF >5% (1.79< OR<3.28 or OR = 0.57). The AUC of the top 10 SNPs ranged from 0.53∼0.6. The top 100 SNPs with lower *p*-values between AERD and ATA are listed in Table S1. Sixty SNPs had *p*-values<0.001 between AERD and ATA. MAFs of these SNPs in subjects with ATA were almost similar to those of the normal controls (Table S1 and Table S2).

**Table 2:** List of 10 SNPs with low *p*-values associated with AERD.

SNPid	Gene(s)	Chr	Allele	Mutation(s)	MAF			AERD vs. ATA		AUC	AERD vs. NC		ATA vs. NC		statisticalpower	HWP
					AERD (n = 165)	ATA (n = 397)	NC (n = 398)	OR (95% CI)	*p*-value		OR(95% CI)	*p*-value	OR(95% CI)	*p*-value		
exm537513*	HLA-DPB1	6	A>C	Missense_I94L	0.167	0.057	0.062	3.28(2.13–5.04)	3.40E-08	0.60	3.20(1.98–5.17)	9.23E-07	1.01(0.66–1.56)	9.60E-01	0.77	0.427
exm537522	HLA-DPB1	6	A>G	Missense_M10V	0.194	0.087	0.085	2.50(1.71–3.67)	2.38E-06	0.59	3.20(1.98–5.17)	9.23E-07	1.07(0.73–1.55)	7.31E-01	0.81	0.372
exm-rs3097671	HLA-DPA1	6	G>C	Silent	0.191	0.088	0.084	2.47(1.69–3.62)	3.19E-06	0.59	2.59(1.68–3.99)	1.05E-05	1.10(0.76–1.60)	6.19E-01	0.81	0.317
exm-rs3129294	HLA-DPB2	6	T>G	Silent	0.324	0.207	0.206	1.91(1.41–2.58)	2.75E-05	0.60	1.85(1.32–2.60)	3.42E-04	0.96(0.74–1.24)	7.59E-01	0.9	0.902
exm83523*	CHIA	1	G>A	Missense_G102R	0.133	0.064	0.065	2.55(1.64–3.95)	3.00E-05	0.56	2.02(1.23–3.31)	4.98E-03	0.91(0.59–1.42)	6.86E-01	0.7	0.039
exm1884673*	THRA	17	A>G	Missense_I170V	0.033	0.003	0.006	13.61(2.89–63.96)	6.20E-05	0.53	5.30(1.40–20.12)	9.39E-03	0.54(0.09–3.18)	4.86E-01	0.28	0.658
exm538564*	ZBTB22	6	T>C	Missense_T310A	0.385	0.273	0.265	1.79(1.33–2.39)	9.25E-05	0.59	2.39(1.69–3.39)	4.51E-07	1.08(0.85–1.39)	5.28E-01	0.85	0.423
exm2264237*	LOC401320	7	A>C	Silent	0.330	0.438	0.436	0.57(0.42–0.76)	1.10E-04	0.58	0.56(0.41–0.77)	3.02E-04	1.03(0.83–1.29)	7.79E-01	0.91	1.000
exm396794*	SLC30A9	4	G>A	Missense_M50V	0.009	0.052	0.040	0.13(0.03–0.53)	1.40E-04	0.54	0.13(0.03–0.60)	9.48E-04	1.50(0.89–2.54)	1.26E-01	0.11	0.906
exm791954*	TSC1	9	T>C	Synonymous_E444E	0.130	0.063	0.062	2.38(1.50–3.76)	2.40E-04	0.56	2.35(1.38–4.02)	1.81E-03	1.05(0.67–1.64)	8.23E-01	0.7	0582

*Indicates the 7 SNPs that were part of a model.

### Multiple logistic regression and ROC curve analysis of the top 10 SNPs

**Figure 3:**
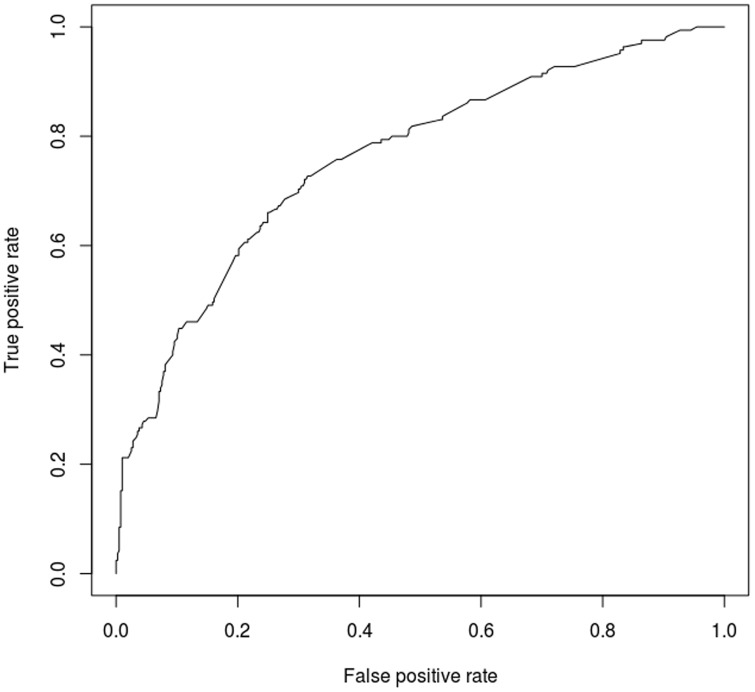
ROC curve of the best AUC model consisting of 7 SNPs.

A multiple logistic regression analysis was undertaken for all power sets composed of the 10 SNPs under the additive mode. ROC curves and the AUC were calculated for the 1,023 (2^10^−1) combinations of the 10 SNPs. The AUC values and *p*-values of the 1,023 models are presented in Table S4. The 192 candidate models of 1,023 models turned out to be that all *p*-values of the coefficients were less than 0.05. One model of 192 models, composed of seven SNPs (exm537513, exm83523, exm1884673, exm538564, exm2264237, exm396794, and exm791954; marked by an ‘*’ in Table 2), had the highest AUC of 0.75 (an asymptotic *p*-value of 7.94×10^−21^ and an asymptotic 95% confidence interval of 0.706−0.795) (Fig. 3).

The results of the contingency using the seven SNPs are presented in Table 3. The combination of these SNPs had 34% sensitivity and 93% specificity to discriminate AERD from ATA. The results showed a positive predictive value of 68.2% and a negative predictive value of 77.1% with an accuracy of 76%. The result of 5-fold cross-validation for the combination model of 7 SNPs (exm537513, exm83523, exm1884673, exm538564, exm2264237, exm396794, and exm791954) showed 21.6–47.2% sensitivity (mean: 30.4%) and 90.7–97.4% specificity (mean: 93.6%), which were comparable to those obtained using multiple logistic regressions.

**Table 3:** The contingency of the seven SNPs that formed the model.

	Actual value		Sensitivity	Specificity
Predicted outcome	ATA	AERD		
ATA	368	110	33%	93%
AERD	29	55		

### Functional implication of SNP alleles and structure effect of the selected seven SNPs

CpG Islands, DNAse I hypersensitive sites, and microRNA target sites were evaluated using the SNP functional Portal (http://brainarray.mbni.med.umich.edu/Brainarray/Database/SearchSNP/snpfunc.aspx). Any of the seven SNPs were not related with these sites. Of the seven exonic SNPs, five were missense SNPs leading to changes of amino acids, or changes in side-chain polarity and charges (Table 4). The exm83523 was predicted to be “probably damaging” with the probability of 1 PloyPhen2 score due to changes in polarity and charge. The exm538564 was predicted to have different polarity from polar to non-polar change. The other three SNPs were predicted no change in either charge or polarity.

**Table 4:** The structural change and amino acid properties change of five missense SNPs.

Exomid	SNPid	Gene	AA Change	Polyphen2	The change of side-chain property	
				Score	Polarity change	Charge change
exm537513	rs1042136	HLA-DPB1	I→L	0	Nonpolar → nonpolar	Neutral → neutral
exm83523	rs3818822	CHIA	G→R	1	Polar → Basic polar	Neutral → positive
exm1884673	rs118191745	THRA	I→V	0	Nonpolar → nonpolar	Neutral → neutral
exm538564	rs3130100	ZBTB22	T→A	0	Polar → nonpolar	Neutral → neutral
exm396794	rs1047626	SLC30A9	M→V	0	Nonpolar → nonpolar	Neutral → neutral

## Discussion

We analyzed the Exome BeadChip data for a total of 562 asthmatics (AERD, 165; ATA, 397) and 398 normal controls. The comparison between the observed and the expected association *p*-values in the Q-Q plot revealed a λ value of 1.0013, indicating the absence of stratification of the study population and the presence of SNPs with distinct deviations from the expected.

A previous GWAS study [31], showed that the *HLA-DPB1* rs1042151 (Met105Val) SNP was most significantly associated with susceptibility to AERD with an OR of 2.40. In the present study, three exonic SNPs on *HLA-DPB1* occurred in the top 20 SNPs: exm537513, exm537522 and exm537523. Exm537513 on *HLA-DPB1* had the lowest *p*-value (3.4×10^−8^; OR, 3.28) and, to the best of our knowledge, is a novel SNP associated with AERD. The exm537513 SNP was not amongst the SNPs in our previous GWAS study [31], even though the MAF was >5% (6% in NC and ATA and 17% in AERD). The exm537522 SNP was the same as *HLA-DPB1* rs1042151 in our previous GWAS data.

Because the AUC value of exm537513 on *HLA-DPB1* was 0.60, with very low sensitivity (3.6%) for predicting AERD, multiple logistic regression was performed using six exonic SNPs in the *HLA* genes (three in *HLA-DPB1*, two in *HLA-DPA1* and one in *HLA-DPB2*) present in the top 20 exonic SNPs. The combination of four SNPs (exm537513, exm537522, exm-rs3097671, and exm-rs3129294) raised the AUC to 0.629, although the combination of all six SNPs showed an AUC of 0.625. This may be due to the strong LD between exm537485 and exm537522 (D’ = 1) and between exm537522 and exm537523 (D’ = 1), as shown in Table S2, since they are located very close to each other; 204bps between exm537485 and exm537522 and 2bps between exm537523 and exm537522.

To further enhance the prediction of AERD, the six SNPs were combined with the four SNPs in the *HLA* gene, and the AUC was calculated for the 1,023 (2^10^−1) combinations. The greater the number of SNPs used for the analysis, the higher the possible predictive combination. However, combinations of more than 10 SNPs were impractical. Here, one model composed of seven SNPs showed the highest AUC of 0.75, representing a novel combination to predict AERD. To validate the best prediction model of the 7 SNPs obtained by using multiple logistic regressions, 5-fold cross-validation was applied. The sensitivity and specificity to discriminate AERD from ATA were comparable between the two methods. This data suggests that the best prediction of combination model of 7 SNPs seem to be reliable. In the present study, predictive modeling for discrimination of AERD from ATA had the high specificity but the low sensitivity. This may be due to small number of AERD compared to ATA patients in this study. When a balanced sample (AERD: 165, ATA: 165) is used for the prediction model consisting of the 7 SNPs (exm537513, exm83523, exm1884673, exm538564, exm2264237, exm396794, and exm791954), the prediction of AERD is 67% sensitivity and 73% specificity. The result shows increase in the sensitivity while decrease in specificity more than that of the best model using unbalanced samples (AERD: 165, ATA: 397). Thus, the prediction model in the present study may increase the sensitivity when the more AERD patients are included, and our prediction model may be reliable at the moment.

*HLA-DPB1* has a strong link with asthma, ATA, AERD and inflammation [13], [31], [46]–[48]. To date, 873 SNPs in *HLA-DPB1* and 1,146 SNPs in *HLA-DPA1* have been identified (http://www.ncbi.nlm.nih.gov/snp/?term=HLA-DPB1+and+human, http://www.ncbi.nlm.nih.gov/snp/?term=HLA-DPA1+and+human). Among them, 260 and 205 exonic SNPs are present in *HLA-DPB1* and *HLA-DPA1*, respectively. In the present study, 12 SNPs in the *HLA-DPB1* and 20 SNPs in the *HLA-DPA1* were investigated in association with AERD. This indicates that the number of SNPs on the chip used in the present study is insufficient to cover all SNPs identified to date. This minimal coverage may be a limitation in the identification of AERD- associated rare variants.

Recently, rare variants were analyzed in an association study to detect SNPs with higher impacts on the genetic risk for multi-complex diseases [49]. Among the 54,555 SNPs investigated in the present study, 6,878 SNPs (12.6%) were found to have a MAF<1% in AERD, ATA, or NC. In the top 10 SNPs, *THRA* and *SLC30A9* was rare variants with a frequency <1% in one or two of the study groups. The AUC values of these SNPs were lower than those of the other eight top SNPs, with a MAF>5%. This indicates that rare variants may have less discrimination power than common variants for the prediction of AERD.

*HLA-DP* is an αβ-heterodimer cell-surface receptor. The α-helical domain forms the side of the peptide binding groove. The β-sheet regions form the base of the binding groove, the bulk of the molecule, as well as the inter-subunit binding region [50]. In the present study, five SNPs in *HLA-DP2* (three exonic SNPs in *HLA-DPB1* and two exonic SNPs in *HLA-DPA1*) were among the top 20 SNPs. There were four missense SNPs, and three of these (exm537513, exm537522, and exm537523) were located in the binding site of the small peptides (Figure S2), with amino acid changes at these loci potentially changing the binding affinity of antigens related to AERD.

The functional relationship of the remaining six top genes associated with AERD has not yet been clarified. Interleukin 13 is induced by *CHIA*, variations of which may lead to asthma susceptibility [51]. *THRA*, also known as nuclear receptor subfamily 1, group A, member 1 (NR1A1), is a nuclear receptor protein [52]. *TSC1* inhibits the nutrient-mediated or growth factor-stimulated phosphorylation of S6K1 and EIF4EBP1 by negatively regulating mTORC1 signaling [53]. The function of the *ZBTB22, LOC401320* and *SLC30A9* genes has not been identified in the PubMed database (http://www.ncbi.nlm.nih.gov/pubmed).

The SNP functional Portal was used to find out the effect on the transcription of the selected seven SNPs for CpG Island, DNAse I hypersensitive site, microRNA target site. The result showed that they had no effect on gene expression. The expected impacts of five missense site of the seven exonic SNPs were evaluated on the proteins structure and function. Among them, exm83523 *CHIA* showed changes in side-chain polarity (polar → Basic polar) and in side-chain charge (neutral → positive) showing the score of “probably damaging”. This data suggest that exm83523 *CHIA* might have the greatest impact on the function of *CHIA* protein.

We have previously reported the results of a 600K GWAS in association with the risk of AERD [54]. When the nine genes of the top 10 SNPs in the present exome study were searched among the top 100 genes of the 600K GWAS data (Table S5), only two genes (*HLA-DPB2* and *TSC1*) were identified. This may be due to a minimal overlap of the 600K GWAS and the Exome BeadChip. There was an overlap of only 9,932 SNPs between the Exome BeadChip (4.09% of 242985 SNPs) and the GWAS chip (2.31% of 430487 SNPs). Another limitation of this study was as follows. First, no replication study was performed, and the number of study subjects was relatively small to detect significance of rare variants. Second, a functional study for the candidate SNPs was not performed. Therefore, a follow-up study for the replication and a functional study of the candidate SNPs would be needed to clarify a relationship between the SNPs and the development of AERD.

## Supporting Information

Figure S1 **The result of the MDS (multidimensional scaling) plot analysis for the population substructure.**
(TIF)

Figure S2 **The location of four missense SNPs on HLA-DPA1 and HLA-DPB1 protein structure: rs1126504, rs1042136, rs1042151 and rs1042153.**
(TIF)

Table S1 **The top 100 SNPs with lower** ***p*** **-values between AERD and ATA.**
(XLS)

Table S2 **Linkage disequilibrium (LD) between the six exonic SNPs in the HLA genes.**
(XLS)

Table S3 **The AUCs of combinations (2^6^−1) for the six exonic SNPs in the HLA genes.**
(XLS)

Table S4 **The AUCs of combinations (2^10^−1) for the top 10 candidate SNPs.**
(XLS)

Table S5 **Comparison between top nine genes in Exome data and top 100 genes in GWAS data.**
(XLS)
