# Supplementary figures and images for: Exonic Variants Associated with Development of Aspirin Exacerbated Respiratory Diseases
Source: PLoS One. 2014 Nov 5;9(11):e111887. doi: 10.1371/journal.pone.0111887 (PMC4221198; doi:10.1371/journal.pone.0111887)

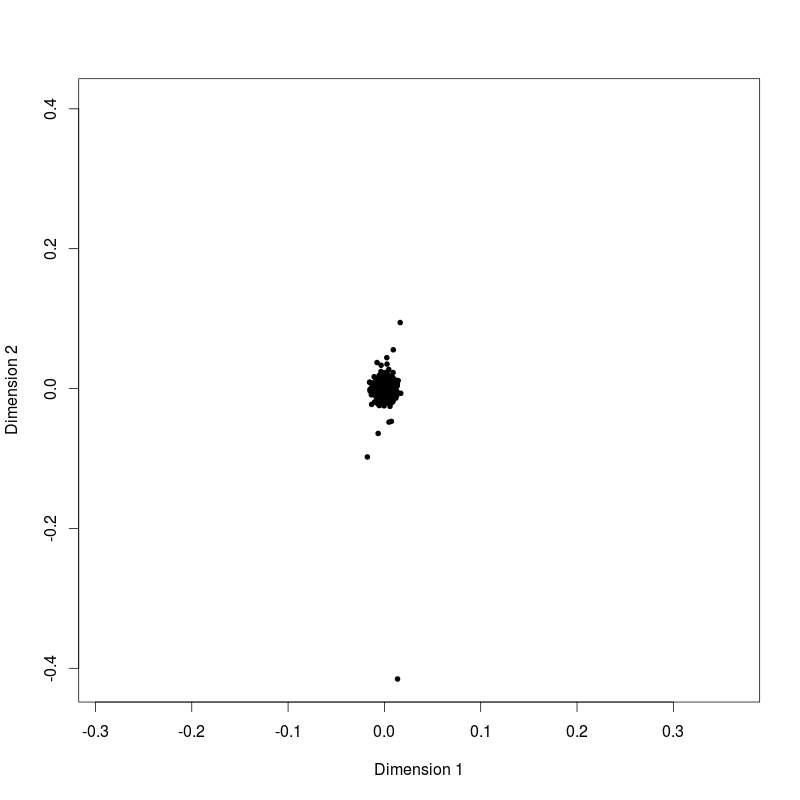

Supplement: Figure S1 — The result of the MDS (multidimensional scaling) plot analysis for the population substructure. (TIF) [file pone.0111887.s001.tif]

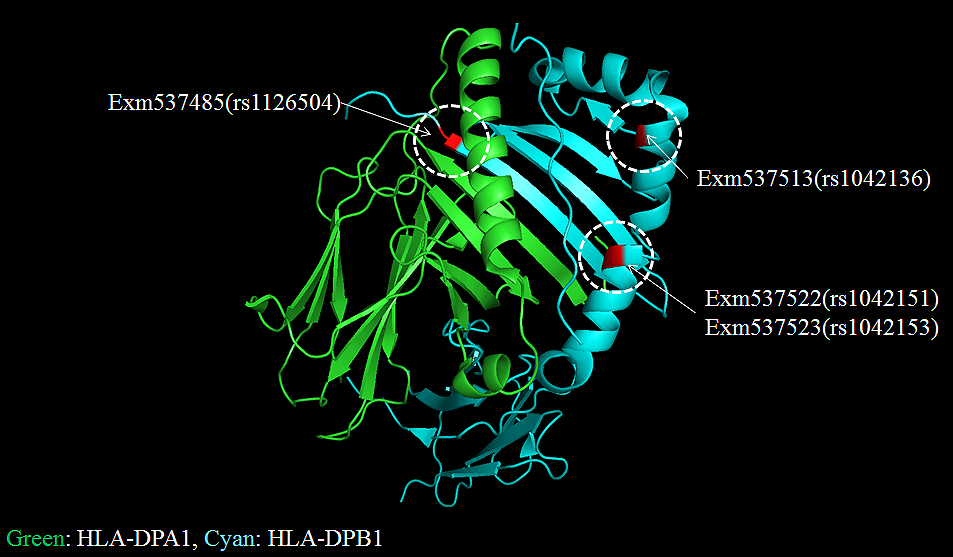

Supplement: Figure S2 — The location of four missense SNPs on HLA-DPA1 and HLA-DPB1 protein structure: rs1126504, rs1042136, rs1042151 and rs1042153. (TIF) [file pone.0111887.s002.tif]
